# Association of Circulating Omentin-1 with Osteoporosis in a Chinese Type 2 Diabetic Population

**DOI:** 10.1155/2020/9389720

**Published:** 2020-10-15

**Authors:** Pijun Yan, Yong Xu, Zhihong Zhang, Jianhua Zhu, Ying Miao, Chenlin Gao, Qin Wan

**Affiliations:** ^1^Department of Endocrinology, The Affiliated Hospital of Southwest Medical University, Luzhou, Sichuan 646000, China; ^2^Department of General Medicine, The Affiliated Hospital of Southwest Medical University, Luzhou, Sichuan 646000, China

## Abstract

**Aims:**

Omentin-1, a newly identified adipokine, has been demonstrated to be associated with bone metabolism, but the results have been inconsistent. Moreover, the potential relationship of circulating omentin-1 with diabetic osteoporosis has never been reported. This study is intended for studying the association between circulating omentin-1, bone mineral density (BMD), prior fragility fractures, and other bone metabolic-related parameters.

**Methods:**

Circulating omentin-1 levels were measured in 172 patients with type 2 diabetes mellitus (T2DM), and participants were divided into the normal BMD group (**n** = 52), the osteopenia group (**n** = 66), and the osteoporosis group (**n** = 54). The relationship between circulating omentin-1 and diabetic osteoporosis and other parameters was analyzed.

**Results:**

Circulating omentin-1 was significantly higher in the osteoporosis group than in the normal group and in the osteopenia group (both **P** < 0.05). Circulating omentin-1 levels were correlated significantly and positively with sex; high-density lipoprotein cholesterol; apolipoprotein A; and prevalence of prior fragility fractures, diabetic nephropathy, and retinopathy; they were correlated negatively with diastolic blood pressure, triglyceride, hemoglobin, atherogenic index of plasma, osteoporosis self-assessment tool for Asians, BMD at different skeletal sites, and corresponding **T** scores, irrespective of age, sex, and body mass index (**P** < 0.01 or **P** < 0.05). Moreover, circulating omentin-1 was an independent decisive factor for the presence of osteoporosis only in women after multivariate adjustment (odds ratio: 1.069; 95% confidence interval: 1.003-1.139; **P** < 0.05). Lastly, the analysis of receiver operating characteristic curves revealed that the best cutoff value for circulating omentin-1 to predict diabetic osteoporosis was 15.37 ng/mL (sensitivity: 71.7%; specificity: 58.5%) in female subjects.

**Conclusions:**

High levels of circulating omentin-1 may be associated with the development of osteoporosis in female diabetic subjects and may be a potential biomarker for diabetic osteoporosis in women.

## 1. Introduction

Type 2 diabetes mellitus (T2DM) is a chronic metabolic disease characterized by an elevated level of blood glucose and insulin resistance (IR), leading to microvascular disease and macrovascular complications. Recent studies have revealed that osteoporosis, also a common diabetic-related complication, is rapidly on the rise, affecting nearly 75 million people worldwide and causing more than 2 million fractures annually [1]. Therefore, diabetic osteoporosis has emerged as a serious global burden upon human health and economic development. The pathogenic mechanisms underlying diabetic osteoporosis remain poorly understood, although several mechanisms, including genetics, estrogen deficiency, IR, dyslipidemia, hyperglycemia, cardiometabolic risk factors, angiopathy, inflammation, oxidative stress, and abnormal cytokine production, have been proposed to be involved in the development of diabetic osteoporosis [2, 3]. Currently, there are few effective therapies for diabetic osteoporosis. It is therefore an urgent task to seek clinically suitable surrogate markers of osteoporosis for preventing and treating diabetic osteoporosis at an early stage.

Recently, adipose tissue has been reported to play critical roles in the regulation of bone biology and remodeling. Abundant evidence in vitro and in vivo has demonstrated that adipose tissue-derived adipokines, including leptin, adiponectin, visfatin, or apelin, could play a key role in the regulation of skeletal health and bone mass [2, 4]. Omentin-1, a novel 34 kDa adipokine predominantly derived from visceral omental adipose tissue, is highly abundant in human plasma [5, 6]. Several lines of experimental and clinical evidence have shown that omentin-1 can modulate energy metabolism, promote insulin-mediated glucose transport, and has insulin-sensitizing, anti-inflammatory, antiatherogenic, and cardioprotective properties. Circulating omentin-1 was associated with inflammatory disease, obesity, IR, diabetes and diabetic vascular complications, hypertension, hyperlipidemia, metabolic syndrome, polycystic ovary syndrome, obstructive sleep apnea syndrome, chronic obstructive pulmonary disease, endothelial dysfunction, and atherosclerosis and related cardiovascular diseases such as ischemic heart disease [6–11], all of which have been proposed to be implicated in the pathogenesis of osteoporosis [3, 12, 13]. Moreover, it has been reported that serum omentin-1 inversely correlates with leptin and positively correlates with adiponectin and vitamin D [7, 9, 14], whereas the abovementioned cytokines have also been suggested to be involved in bone metabolism [14]. Thus, we postulated that omentin-1 may be associated with the regulation of bone metabolism and might play a pivotal role in the pathogenesis of osteoporosis. In line with the hypothesis, it is suggested that omentin-1 may play pivotal roles in the regulation of bone metabolism, but the results have been inconsistent. Several studies showed that high omentin-1 protected from bone mass loss and osteoporosis by suppressing bone resorption and promoting bone formation in mice and in multiple sclerosis patients [15, 16]. Other studies showed that omentin-1 has a negative effect on bone mass at different skeletal sites, and on bone turnover markers through inhibiting bone formation in Chinese premenopausal women, Iranian postmenopausal women, girls with anorexia nervosa, and older men; other studies showed that omentin-1 has no effect in both postmenopausal and premenopausal women and in Chinese healthy males [4, 8, 14, 17, 18, 19, 20]. Based upon the evidences outlined above, omentin-1 may exert ambiguous effects on bone mass, which may be caused by the small sample sizes, different study populations and metabolic characteristics, and complex regulatory mechanisms in vivo. Moreover, the relationship between circulating omentin-1 and bone mineral density (BMD) and osteoporosis in patients with T2DM has never been investigated yet.

Therefore, the objective of the present study was to evaluate the association of circulating omentin-1 with osteoporosis assessed by BMD and verified self-reported fragility fractures in T2DM subjects. The possible relation of circulating omentin-1 with cardiometabolic parameters and inflammatory markers was also investigated.

## 2. Methods

### 2.1. Study Population

A total of 172 patients with T2DM aged 45–89 years, recruited from our inpatient department between June 2015 and January 2017, were finally enrolled in the cross-sectional study. All patients had a diagnosis of T2DM according to an oral glucose tolerance test and the American Diabetes Association criteria in 1997 [21]. Inclusion criteria were (1) confirmed or newly diagnosed T2DM patients, (2) postmenopausal women aged ≥45 years who had not menstruated for at least 1 year or men aged ≥50 years, and (3) long-term residence (≥5years) in China's Sichuan province. The exclusion criteria were type 1 diabetes mellitus, acute complications of diabetes, thyroid diseases, hyperparathyroidism, hypothalamic and pituitary diseases, hypogonadism, Cushing syndrome, cardiac arrhythmias, including atrial fibrillation, aortic stenosis, myocardial infarction or unstable angina within the previous 3 months, uncontrolled hypertension > 180/100 mmHg, acute respiratory failure, asthma, chronic obstructive pulmonary disease, chronic liver disease, thromboembolic disease, hematological system diseases, acute or chronic infection, inflammatory disease, autoimmune disease such as Behcet's disease, chronic periodontitis, psoriasis, psoriatic arthritis, rheumatoid arthritis, ankylosing spondylitis, Kawasaki disease, and synovitis, gastrointestinal system diseases such as Crohn's disease, ulcerative colitis, and pancreatitis, alcohol abuse, cigarette smoking (former or current), inability to ambulate, impaired cognitive function, malignancies, pregnancy or lactation, any other etiological cause of fractures (e.g., systemic glucocorticoids, cancer, traffic accidents, high-trauma, and family history), and use of drugs that might alter bone metabolism for more than 6 months or within the previous 12 months, such as thiazolidinedione, sodium-dependent glucose transporter 2 inhibitors, calcium supplements, vitamin D, calcitonin, bisphosphonates, teriparatide, and estrogens.

The study was performed in accordance with the ethical guidelines of the 1975 Declaration of Helsinki and was reviewed and approved by the human research ethics committee of the Affiliated Hospital of Southwest Medical University, and all the patients provided informed consent before taking part in the study.

### 2.2. Anthropometric and Biochemical Measurements

All subjects completed a standard questionnaire for collecting data about diabetic duration, lifestyle habits (alcohol consumption and cigarette smoking), previous or current diseases (hypertension, stroke, coronary heart disease (CHD), peripheral arterial disease (PAD), diabetic retinopathy (DR), diabetic nephropathy (DN) and diabetic peripheral neuropathy (DPN), and other diseases), and use of medications, and all subjects underwent a comprehensive physical examination according to standard procedures.

Body weight and height were measured with the subject wearing light clothes without shoes, and body mass index (BMI) was then calculated. Blood pressure was measured on the right arm using a standard mercury sphygmomanometer in the supine position after 10 min of rest.

Blood samples were obtained from all individuals in early morning following overnight fasting for at least 8 h. A prespecified volume of blood samples was centrifuged at 3500 rpm for 10 min at 4°C and filled into 1 mL aliquots and stored at -80°C until analyzed. Fasting blood glucose (FBG), glycated hemoglobin A1C (HbA1c), lipid profiles, including total cholesterol (TC), triglyceride (TG), high-density lipoprotein cholesterol (HDL-C), low-density lipoprotein cholesterol (LDL-C), apolipoprotein A (apoA), apolipoprotein B (apoB), total bilirubin (TBIL), direct bilirubin (DBIL), indirect bilirubin (IBIL), gamma-glutamyltransferase (GGT), alkaline phosphatase (ALP), serum calcium and phosphorus ion, creatinine, hemoglobin (Hb), white blood cell (WBC), neutrophil, and lymphocyte counts, neutrophil to lymphocyte ratio (NLR), 25-hydroxyvitamin D (25(OH)D), and intact parathyroid hormone (PTH) were measured according to relevant protocols and guidelines at a certified laboratory. Plasma calcium ion was adjusted for hypoalbuminemia as previously reported. Corrected calcium concentration (mg/dL) = serum calcium concentration (mg/dL)+0.8×[4.0 (g/dL−serum albumin concentration (g/dL)] Urinary microalbumin and creatinine were measured, and the urinary albumin-to-creatinine ratio (ACR; mg/g creatinine) was calculated, as we described previously [22]. Renal function expressed as the estimated glomerular filtration rate (eGFR) was calculated using Chronic Kidney Disease Epidemiology Collaboration (CKD-EPI) equations modified by a Japanese coefficient [22]. Patients were then classified as having DN if they had an eGFR < 60 mL/min/1.73 m^2^ and/or an ACR > 30 mg/g [22].

Osteoporosis Self-Assessment Tool for Asians (OSTA) index can be calculated using the formula of (weight in kilograms − age in years) × 0.2 [23].

Ankle-brachial index (ABI) was measured by a continuous-wave Doppler ultrasound probe (Vista AVS, Summit Co.) in all T2DM patients. PAD was defined as ABI < 0.90 [24].

A vibration perception threshold (VPT) was assessed at the metatarsophalangeal joint dig I using a neurothesiometer (Bio-Thesiometer; Bio-Medical Instrument Co., Newbury, OH, USA). Sensibility to touch was tested using a 10 g Semmes-Weinstein monofilament at four points on each foot: three on the plantar and one on the dorsal side. DPN was defined as VPT ≥ 25 V and/or inability to feel the monofilament [22].

### 2.3. Measurements of Plasma Omentin-1 and Tartrate-Resistant Acid Phosphatase 5b

Circulating levels of omentin-1 (CUSABIO, Wuhan, China; Catalog No. CSB-E09745h) were determined with a commercially available enzyme-linked immunosorbent assay (ELISA), with a sensitivity of 0.39 pg/mL and a reference range of 1.56-100 pg/mL. Plasma tartrate-resistant acid phosphatase-5b (TRAP-5b) (CUSABIO, Wuhan, China; Catalog No. CSB-E08490h) was also detected by using a human ELISA kit, with a sensitivity of 0.078 mIU/mL and a reference range of 0.312–20 mIU/mL, according to the protocol. The inter- and intra-assay coefficients of variation (CVs) for omentin-1 and TRAP5b were less than 8% and 10%, respectively.

### 2.4. BMD Measurement and Diagnostic Criteria of Osteoporosis

The areal BMD values at the lumbar spine (LS), femoral neck (FN), and total hip (TH) were captured by dual-energy X-ray absorptiometry (DXA) using the GE Lunar Prodigy and were expressed as g/cm^2^, as well as in **T** scores (deviation from the peak BMD). All measurements were taken by the same well-trained and qualified operator on the same machine using standardized protocols for participant positioning to ensure machine accuracy of >98%. The CVs of measurement of BMD at the LS, FN, and TH in our lab were 0.84%, 1.96%, and <1.72%, respectively.

All participants were asked whether there was a history of fractures, including the time, site, and cause of fractures. Fragility fractures included fractures of axial (ribs, lumbar, and thoracic vertebrae) and peripheral bones (forearm, humerus, and femur) that resulted from minimal or moderate trauma [13]. Self-reported fragility fractures were included in analyses if verified by clinical symptoms, physical examination, medical and hospital records, and radiographs or computed tomography or magnetic resonance imaging or other methods such as whole body bone scan. Traumatic fractures and those occurring at sites not characteristic of bone fragility (face, skull, tibia, fibula, and femoral diaphysis) were excluded from the analysis [13].

According to the WHO guidelines, osteopenia that denoted a **T** score of -1 to -2.5 SD and a **T** score of -2.5 SD or lower at any of the sites on the LS, FN, and TH indicates osteoporosis [24, 25]. In our present study, the osteoporotic patients with and without verified self-reported fragility fractures and osteopenic patients with verified self-reported fragility fractures were diagnosed as having diabetic osteoporosis [24]. All participants were subsequently divided into three groups: the normal group (**n** = 52), the osteopenia group (**n** = 66), and the osteoporosis group (**n** = 54) according to the **T** score and a history of fragility fractures [24].

### 2.5. Statistical Analysis

All analyses were performed with the Statistical Package for Social Sciences version 20.0 (SPSS, Chicago, IL). First, Kolmogorov-Smirnov's test for normality and Levene's homogeneity of variance test were conducted. Data are expressed as mean ± SD for continuous variables or numbers (percentages) for categorical variables.

Differences among more than three or more groups were evaluated with one-way analysis of variance (ANOVA) followed by the Least Significant Difference (LSD) post hoc test for multiple comparisons (continuous variables with normal distribution and homogeneity of variance), or the Kruskal-Wallis test followed by all pairwise for multiple comparisons (covariates with nonparametric distribution and/or uneven variance). The correlation of circulating omentin-1 and other variables was assessed by Pearson's correlation coefficient or Spearman's rank correlation as appropriate. Partial correlation analyses were used to extract the correlation coefficient after adjusting for age, sex, and BMI. Then, the univariate and multivariable logistic regression analyses were performed to determine the association of circulating omentin-1 and other variables with risk of osteoporosis in all study subjects, male subjects, and female subjects, respectively. Odds ratios (OR) and 95% confidence intervals (CI) were estimated. Lastly, receiver operating characteristic (ROC) curve analysis was performed to determine the optimal cutoff point of circulating omentin-1 for the diagnosis of osteoporosis in all study subjects, male subjects and female subjects, respectively.

All **P** values are two-tailed, and values of less than 0.05 were considered to indicate statistical significance.

## 3. Results

### 3.1. Circulating Levels of Omentin-1 and Other Clinical Characteristics of the Studied Population

**Figure 1:**
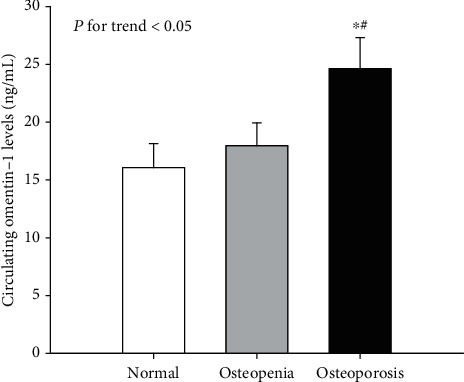
Circulating levels of omentin-1 among the three groups. Versus the normal group: ∗*P* < 0.05. Versus the osteopenia group: ^#^*P* < 0.05.

The general characteristics of the studied population are summarized in Table 1. There were significant differences in gender distribution, age, height, weight, Hb, TBIL, IBIL, GGT, serum albumin, eGFR, urinary ACR, ABI, omentin-1, OSTA, and BMD at the LS, FN, and TH and corresponding **T** score, and prevalence of prior fragility fractures, PAD, and DN among the three groups. When compared with those with normal BMD and osteopenia, T2DM patients with osteoporosis had significantly more female subjects, higher circulating omentin-1 and prevalence of prior fragility fractures, and lower height, weight, ABI, OSTA, and BMD at different skeletal sites and corresponding **T** scores (**P** < 0.01 or **P** < 0.05; Table 1 and Figure 1). They also were significantly older; had a higher level of urinary ACR; had a prevalence of DN; had lower Hb, TBIL, IBIL, GGT, and eGFR compared with individuals with normal BMD; and had a higher prevalence of PAD than individuals with osteopenia (**P** < 0.01 or **P** < 0.05; Table 1).

### 3.2. Association of Circulating Omentin-1 with Anthropometric, Biochemical, and Clinical Parameters in Study Subjects

**Figure 2:**
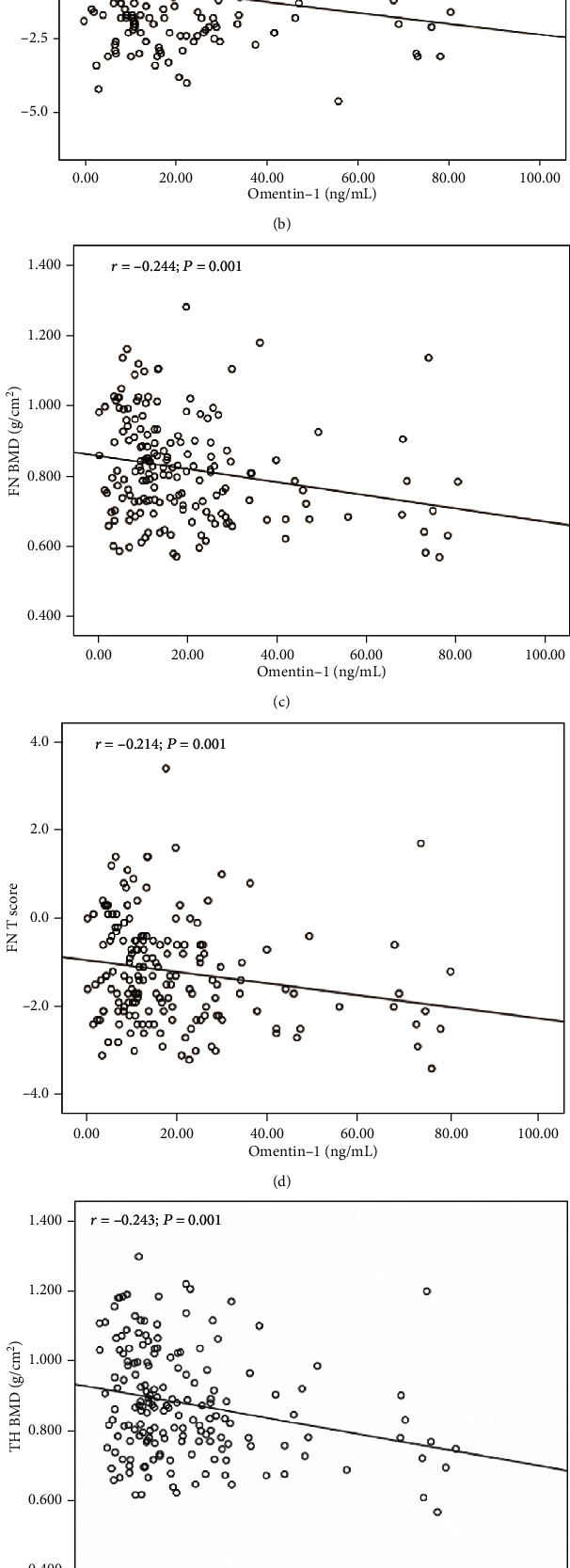
Correlations between circulating omentin-1 and BMD at different skeletal sites and corresponding *T* scores. (a) LS BMD, (b) LS *T* score, (c) FN BMD, (d) FN *T* score, (e) TH BMD, and (f) TH *T* score in patients with all study subjects.

Next, we evaluated the association between circulating omentin-1 and various other parameters by using bivariate correlation analyses. In T2DM patients, circulating omentin-1 was positively associated with sex; HDL-C; apoA; NLR; urinary ACR; and prevalence of prior fragility fractures, DN, and DR, and it was negatively associated with TG, lymphocyte count, Hb, TBIL, DBIL, GGT, UA, OSTA, and BMD at different skeletal sites and corresponding **T** scores (**P** < 0.01 or **P** < 0.05; Table 2 and Figure 2). Partial correlation analysis after controlling for age, sex, and BMI revealed that circulating omentin-1 was significantly and positively correlated with HDL-C; apoA; and prevalence of prior fragility fractures, DN, and DR, and it was negatively correlated with DBP, TG, lymphocyte count, Hb, OSTA, and BMD at different skeletal sites and corresponding **T** scores (**P** < 0.01 or **P** < 0.05; Table 2).

### 3.3. Multivariable-Adjusted ORs for the Association of Circulating Omentin-1 with Increased Presence of Osteoporosis in All Study Subjects

To assess whether circulating omentin-1 can reduce the risk of development of osteoporosis, univariate and multivariate logistic regression analyses were mapped. As shown in Table 3, univariate logistic regression analysis revealed that Hb, TBIL, GGT, and serum albumin were negative predictors, whereas sex, age, omentin-1, and prevalence of DN were positive predictors of the presence of osteoporosis. In addition, the presence of osteoporosis tended to be positively associated with ALP and prevalence of PAD (**P** = 0.052 and **P** = 0.057), and negatively related to BMI (**P** = 0.061). Importantly, circulating omentin-1 remained an independent predictor for osteoporosis after multivariable adjustment (odds ratio: 1.035; 95% confidence interval: 1.001-1.070; **P** < 0.05).

### 3.4. Circulating Omentin-1 Level and Its Association with the Increased Presence of Osteoporosis in Men and Women, Respectively

When compared with male subjects, female subjects had significantly higher circulating omentin-1 levels (**P** < 0.01; Table 4). Logistic regression analysis revealed that circulating omentin-1 levels were significantly associated with the presence of osteoporosis only in women, independent of potential confounding variables (**P** < 0.05; Table 4).

### 3.5. The Predictive Value of Circulating Omentin-1 in Detecting Osteoporosis

**Figure 3:**
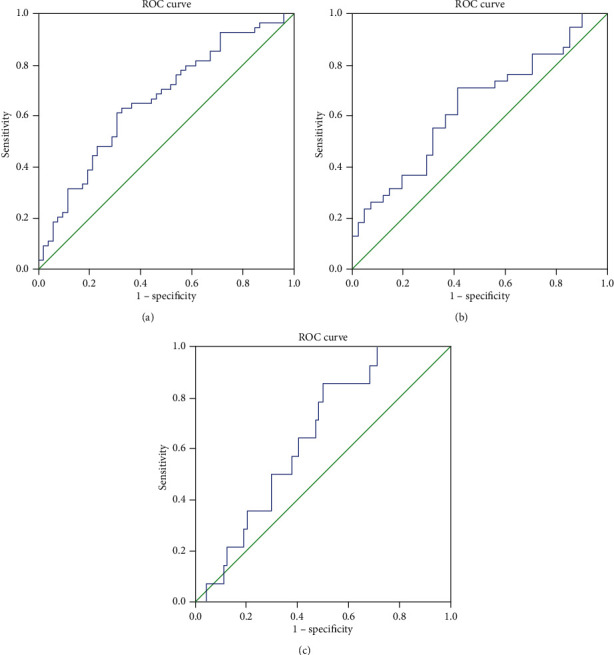
(a) ROC analysis of circulating omentin-1 to indicate osteoporosis for all subjects. AUC = 0.661; 95% CI: 0.558–0.765; *P* = 0.004; identified omentin − 1 cutoff value = 15.28 ng/mL; Youden index = 0.284; sensitivity: 63.0%; specificity: 67.3%. (b) ROC analysis of circulating omentin-1 to indicate osteoporosis for female subjects. AUC = 0.634; 95% CI: 0.511–0.757; *P* = 0.040; identified omentin − 1 cutoff value = 15.37 ng/mL; Youden index = 0.296; sensitivity: 71.1%; specificity: 58.5%. (c) ROC analysis of circulating omentin-1 to indicate osteoporosis for male subjects. AUC = 0.654; 95% CI: 0.524–0.784; *P* = 0.068.

To explore the predictive value of circulating omentin-1 for osteoporosis, we analyzed the ROC curves of circulating omentin-1. The results revealed that the best cutoff value for circulating omentin-1 to predict osteoporosis was 15.28 ng/mL (sensitivity: 63.0%; specificity: 67.3%; and AUC: 0.661) in all subjects (Figure 3(a)), and the best cutoff value for circulating omentin-1 to predict osteoporosis was 15.37 ng/mL (sensitivity: 71.1%; specificity: 58.5%; and AUC: 0.634) in female subjects (Figure 3(b)).

## 4. Discussion

To date, this is the first study to explore the relationship between circulating omentin-1 and the risk of osteoporosis in T2DM patients. We found that circulating omentin-1 in the osteoporosis group was significantly increased compared to those in the normal and osteopenia groups. We also found that circulating omentin-1 correlated positively with the prevalence of prior fragility fractures but associated negatively with BMD at different skeletal sites and corresponding **T** scores and OSTA. This could be used conservatively to identify individuals who are likely to have low BMD and be classified as at risk of having osteoporosis [23]. Moreover, circulating omentin-1 was an independent decisive factor for the presence of osteoporosis only in women after multivariate adjustment. Lastly, circulating omentin-1 was found to only predict the presence of diabetic osteoporosis in women. These findings suggest that circulating omentin-1 may be a useful biomarker of osteoporosis, and high circulating omentin-1 concentration may be associated with an increased risk of female diabetic osteoporosis.

Omentin-1, a newly identified adipokine in human visceral omental adipose tissue, is extensively involved in the regulation and maintenance of a wide variety of physiological and pathological processes, including insulin sensitivity, energy expenditure, blood pressure, glycolipid metabolism, inflammatory response, neuroendocrine activity, immunity, homeostasis, angiogenesis, endothelial and cardiovascular function, reproduction, and recently bone metabolism [6, 7, 11]. In the present study, we firstly found that T2DM patients with osteoporosis had significantly higher circulating omentin-1. We also found that circulating omentin-1 levels correlated significantly and positively with the prevalence of prior fragility fractures and negatively with BMD at different skeletal sites and corresponding **T** scores, and OSTA. Our results are similar to those of previous studies [4, 11, 14, 17]. Wang et al. demonstrated that omentin-1 levels appeared to be significantly higher in pre- compared to postmenopausal women. They also demonstrated that and omentin-1 negatively correlated with bone formation markers, bone-specific alkaline phosphatase (BAP), and bone cross-linked N-terminal telopeptides of type I collagen (NTX), and that it was an independent negative predictor of BMD at different skeletal sites only in premenopausal Chinese women [17]. Moreover, Gołąbek et al. and Guo et al. found significant negative correlations between serum omentin-1 and BMD at the LS and TH as well as bone formation (BAP, osteocalcin (OC), the OC/C-terminal telopeptide fragments of type I collagen (CTX), and osteoprotegerin (OPG)/soluble receptor activator of nuclear factor-*κ*B ligand (sRANKL) ratios) and resorption markers (CTX and NTX) in girls with anorexia nervosa [11, 14, 26]. A similar correlation of circulating omentin-1 levels with BMD at the LS was also found in healthy Iranian postmenopausal women [4]. Interestingly, previous studies have reported an inverse relationship of adiponectin (as a good adipocytokine) with BMD [6, 18, 27], while omentin-1 as a good adipocytokine has similar features and functions to adiponectin. Therefore, both our hereby presented findings and the results of previous studies seem to suggest that there may be a potential mechanistic association between higher circulating omentin-1 and the development of diabetic osteoporosis, and circulating omentin-1 may be upregulated to compensate for bone remodeling enhancement in response to lower BMD caused by cardiometabolic risk factors, inflammation, or vascular damage in T2DM patients. This hypothesis is supported by the report of Dikker et al., which found that omentin-1 levels were significantly increased in postmenopausal women with osteoporosis compared with premenopausal women, and omentin-1 levels positively correlated with vitamin D [8]. Moreover, circulating omentin-1 was an independent decisive factor for the presence of osteoporosis only in women after multivariate adjustment. Furthermore, circulating omentin-1 was found to only predict the presence of female diabetic osteoporosis. Taken together, these findings indicate that omentin-1 may act as a protective adipokine in bone mass under diabetic conditions, and the observed higher circulating omentin-1 in female patients with diabetic osteoporosis might be a physiological compensation and adaptation to protect bone from osteopenia; however, the exact mechanism was unclear.

Experimental and epidemiological studies have shown that chronic low-grade inflammation, evidenced by elevated concentrations of circulating TNF-*α* and IL-6 originally identified in white blood cells, plays a pivotal role in the pathogenesis and progression of osteoporosis by promoting bone resorption through upregulating the RANKL/OPG pathway [3, 12, 13, 28]. Our study provided further evidence that supported the potential role of a dysregulated inflammatory response in the development of diabetic osteoporosis, since we found that T2DM patients with osteoporosis had significantly lower Hb, TBIL, IBIL, and GGT (antioxidative biomarkers); slightly but not significantly lower lymphocyte counts and serum UA and albumin (antioxidative and anti-inflammatory biomarkers); and higher NLR and neutrophil counts (proinflammatory markers) compared with those with normal BMD, and some markers, including TBIL, GGT, serum albumin, and especially Hb, were all predictors of the presence of diabetic osteoporosis. Moreover, we showed that circulating omentin-1 was positively associated with NLR and negatively associated with lymphocyte count, Hb, TBIL, DBIL, GGT, and UA, and the negative association of circulating omentin-1 with lymphocyte count and Hb is irrespective of age, sex, and BMI, consistent with a previous study [29], implying that complex interactions exist among circulating omentin-1 levels and enhanced inflammation and oxidative stress, and there may be a compensation reaction for circulating omentin-1 to inhibit the excessive inflammation and oxidative stress. It was suggested that omentin-1 may play an important role in defense against pathogenic bacteria and inhibition of bacterial translocation, and lower omentin-1 levels in smokers may contribute to increased susceptibility to infection [6, 11, 28]. Several studies have also shown that gene expression and circulating and synovial fluid levels of omentin-1 were significantly decreased in chronic inflammatory diseases such as Crohn's disease, rheumatoid arthritis, and asthma [11], suggesting that omentin-1 might have an inhibitory effect on inflammatory cytokine. Consistently, Zabetian-Targhi et al. demonstrated that serum omentin-1 levels were positively related to anti-inflammatory cytokines IL-13 and IL-4 as products of Th2 cells, and negatively related with inflammatory markers, including IL-6 and TNF-*α*, in obese participants [30]. Pretreatment of endothelium-denuded thoracic aorta of rat with omentin significantly decreased TNF-*α*-mediated phosphorylation of p38, JNK, and expression of VCAM-1 and monocyte adhesion to smooth muscle cells [31]. It also inhibited the TNF-*α*-induced cyclooxygenase- (COX-) 2 expression through the activation of 5′-AMP-activated protein kinase (AMPK)/endothelial nitrous oxide synthase (eNOS)/nitric oxide (NO) pathways in cultured human endothelial cells [6, 11, 32]. It is well known that the eNOS isoform, widely expressed in bone, regulates osteoblast activity and bone formation [12], and the NO derived from the eNOS pathway acts as a mediator of the effects of estrogen in bone [4]. Thus, omentin-1 may inhibit the restorative activity of osteoclasts and prevent bone loss in postmenopausal women. Recently, Wang et al. demonstrated that omentin-1 attenuated lipopolysaccharide- (LPS-) induced oxidative stress and inflammation in macrophages by inhibiting the TLR4/MyD88/NF-*κ*B signaling pathway, and omentin-1 protected macrophages against LPS-induced expression of COX-2 and secretion of prostaglandin E2, and significantly rescued increased intracellular ROS and reduced GSH level induced by exposure to LPS [33]. Collectively, these data revealed that circulating omentin-1 levels might have a compensatory effect on increased inflammation and oxidative stress in patients with diabetic osteoporosis, and a compensatory increase in circulating omentin-1 may exert beneficial effects on bone metabolism by its anti-inflammatory and antioxidative effects [11]; however, further studies are needed to fully elucidate its mechanism of action.

Considerable researches have indicated that diabetic micro- and macroangiopathy associated with atherosclerosis have a negative effect on bone remodeling and bone marrow cell-trafficking, resulting in poor blood supply to bone and balance, increased risk of falls, and subsequently aggravation of bone loss, osteoporosis, and fractures in patients with T2DM [2, 12, 13, 34]. It is well known that ABI is a noninvasive diagnostic biomarker for lower-extremity PAD, and it is also a reliable indicator of atherosclerosis at other vascular sites and macrovascular disease [24]. Microalbuminuria has been recognized for a long time as a hallmark of incipient DN [35], and is regarded nowadays as an indicator of endothelial dysfunction, which is known to play a pivotal role in the pathogenesis of vascular disease associated with atherosclerosis in diabetics [6, 36]. Our study provided further evidence that supported the potential role of diabetic vasculopathy in the development of diabetic osteoporosis, since we found that T2DM patients with osteoporosis had significantly higher urinary ACR, a prevalence of DN, and lower eGFR and ABI compared with individuals with normal BMD. The prevalence of DN was significantly associated with the presence of osteoporosis and the prevalence of PAD tended to be positively associated as well. Moreover, we showed that circulating omentin-1 was positively associated with urinary ACR and the prevalence of DN and DR. The positive association of circulating omentin-1 with the prevalence of DN and DR is irrespective of age, sex, and BMI, consistent with previous studies [11, 37]. Although it is not clear why circulating omentin-1 levels were positively correlated with the prevalence of diabetic microvascular complications (DN and DR), contrary to the previously established antiatherosclerotic, NO-mediated vasodilative, and cardioprotective roles of omentin-1, a possible explanation can be suggested. In response to diabetic microangiopathy caused by inflammation, oxidative stress, endothelial dysfunction, and atherosclerosis, this paradoxical increased level of circulating omentin-1 may be a compensatory response to accentuate bone loss and osteoporosis in patients with T2DM. Generally, recent studies reported that reduced omentin-1 levels were related with atherosclerosis and cardiovascular disease, including carotid atherosclerosis, carotid plaque, coronary artery disease, acute coronary syndrome or stable anginapectoris, ischaemic stroke, and dilated cardiomyopathy [6–11], whereas there were some different findings in these literature [7, 38–40]. Jung et al. found that higher serum omentin levels were associated with a higher prevalence and severity of cardiac autonomic neuropathy, and seemed to be associated with arterial stiffness, as assessed by baPWV in patients with T2DM [38]. Two studies by Yoo et al. have also reported a positive relationship between serum omentin-1 and arterial stiffening in patients with T2DM [39, 40]. Niersmann et al. showed that higher omentin concentration was associated with a higher risk of primary cardiovascular events, stroke, and cardiovascular death after adjustment for multiple cardiovascular risk factors in individuals with diabetes [7]. Given data from our present and previous studies, it appears possible that this paradoxical increase in circulating omentin-1 levels in T2DM subjects might be a mechanism to compensate for the accelerated bone loss and osteoporosis by modulating vascular inflammation and endothelial function, and improving blood supply to the bones, which, however, is not pronounced enough to confer an osteoprotective effect; further research is required to clarify these relationships and elucidate its mechanism of action.

Growing evidence suggests that cardiometabolic risk factors, including female subjects, advanced age, lower weight and BMI, longer diabetic duration, dyslipidemia, hypertension, and hyperglycemia, are critical elements to the pathogenesis of osteoporosis in patients with T2DM [1, 2, 12, 13, 24]. Our findings provided further evidence that T2DM patients with osteoporosis had significantly more female subjects, older age, and lower weight, and tended to have longer diabetic duration, higher SBP and blood lipids except HDL-C, and lower BMI, DBP, FBG, and HbA1c; meanwhile, gender and age were two independent predictors of the presence of osteoporosis, consistent with previous studies [1, 12, 24]. We further demonstrated that circulating omentin-1 was correlated significantly and positively with HDL-C and apoA and correlated negatively with DBP and TG adjustment for age, sex, and BMI, in agreement with previous studies [6, 41, 42, 43]. Several clinical studies have revealed that the levels of omentin-1 in patients with metabolic syndrome, dyslipidemia, and hypertension were reduced, and circulating omentin-1 was negatively correlated with leptin, TG, TC, LDL-C, and blood pressure and positively correlated with adiponectin and HDL-C [6, 10, 11]. Yamawaki et al. demonstrated that omentin directly induced vasodilation of rat isolated blood vessels through endothelium-derived NO [32]. Moreover, their in vivo study demonstrated that intravenously injected omentin acutely inhibited agonist-induced increases of blood pressure in rats [44]. Recently, the same author showed that chronic omentin treatment inhibited monocrotaline-induced pulmonary arterial hypertension in rats via inhibiting vascular structural remodeling and abnormal contractile reactivity [45]. It has been reported that omentin-1 levels can regulate adiponectin and leptine levels, and circulating hyperleptinemia and hypoadiponectinemia lead to endothelial dysfunction, one of the most important pathomechanisms for hypertension, and hyperleptinemia and hypoadiponectinemia are believed to dysregulate blood pressure, resulting in hypertension [46, 47]. The favorable effects of high adiponectin and low leptin on blood lipid levels were also reported [46, 48]. Additionally, plasma HDL-C was decided by the lipase activity of the liver in the peripheral capillary wall, and these factors were subject to insulin regulation [41], suggesting an effect of circulating omentin-1 on HDL-C through the regulation of insulin. Taken together, these results suggest that circulating omentin-1 level was associated with dysregulated blood pressure and lipids, and may play an important role in the development of diabetic osteoporosis via regulating blood pressure and lipids; however, the mechanism is still largely unknown. A more detailed understanding of the molecular mechanism of omentin-1 action will undoubtedly help to gain further insights into the relationships between omentin-1, dysregulated blood pressure and lipids, other adipokines, and diabetic osteoporosis.

The present study has several limitations that must be considered. First, the cross-sectional design precluded conclusions on the temporal relationship of elevated circulating omentin-1 with diabetic osteoporosis. Thus, further large-scale longitudinal studies are needed, especially to determine the role of circulating omentin-1 in the development of diabetic osteoporosis. Second, imaging examination was not performed in all patients with T2DM. Thus, highly prevalent nonsymptomatic vertebral fractures cannot be ruled out, which will likely underestimate the rule prevalence of diabetic osteoporosis and fractures. Remarkably, though, self-report of previous physician diagnosis is often used to assess the rate of osteoporosis, fractures, and/or diabetes in large-scale population studies. We were also unable to distinguish between different types of fractures, given the small number of people for each type of fracture. Third, we only evaluated leukocytes and its subtypes as inflammation markers, and Hb, bilirubin, UA, GGT, and albumin as oxidative stress parameters. The lack of classical inflammatory and oxidative stress markers such as TNF-*α*, IL-6, glutathione, superoxide dismutase, or 8-iso-prostaglandin F2*α* analysis and/or correlation makes it difficult to draw any consistent conclusion regarding the possibility of these indicators as mentioned above to predict evaluated circulating omentin-1 and the development of diabetic osteoporosis. Fourth, although many potential confounding variables were included in the analysis, it is possible that not all risk factors may have been adequately captured in our study. Despite these limitations, the current study is not without strengths, including a relatively large sample size, the use of a standardized method at a single center, and thorough adjustment for possible confounders, which can raise the reliability of our findings. Moreover, our study, to our knowledge, provides the first clinical evidence on a potential link between circulating omentin-1 and diabetic osteoporosis.

In summary, our study demonstrated that circulating omentin-1 level is significantly elevated in patients with diabetic osteoporosis, and it is an independent predictor of the presence of female diabetic osteoporosis, thereby suggesting that circulating omentin-1 may be a potential biomarker for osteoporosis in female patients with T2DM. However, further prospective, large-scale, randomized controlled studies are warranted to establish our results and elucidate the underlying mechanism of the association between circulating omentin-1 and diabetic osteoporosis.

## Figures and Tables

**Table 1:** Clinical and biochemical characteristics of the studied population.

	Normal (**n** = 52)	Osteopenia (**n** = 66)	Osteoporosis (**n** = 54)	**P**
Male/female	41/11	34/32^∗^	14/40^∗∗^^,#^	0.0001
Diabetic duration (years)	8.68 ± 6.66	10.82 ± 7.75	9.06 ± 6.47	0.206
Age (years)	61.12 ± 8.98	64.45 ± 9.80	67.78 ± 8.56^∗∗^	0.001
Height (cm)	164.96 ± 5.92	158.60 ± 8.78^∗∗^	153.43 ± 8.06^∗∗^^,##^	0.0001
Weight (kg)	71.33 ± 10.85	62.97 ± 10.33^∗∗^	58.43 ± 9.83^∗∗^^,#^	0.0001
BMI (kg/m^2^)	26.17 ± 3.35	25.04 ± 3.73	24.81 ± 3.84	0.124
SBP (mmHg)	136.50 ± 21.31	137.86 ± 22.54	140.30 ± 21.01	0.658
DBP (mmHg)	76.04 ± 14.24	73.89 ± 10.90	74.91 ± 12.76	0.655
TC (mmol/L)	4.43 ± 1.32	4.34 ± 1.12	4.65 ± 1.34	0.390
TG (mmol/L)	1.85 ± 1.28	2.02 ± 1.53	2.51 ± 2.40	0.146
HDL-C (mmol/L)	1.21 ± 0.47	1.13 ± 0.32	1.09 ± 0.29	0.261
LDL-C (mmol/L)	2.64 ± 1.09	2.68 ± 0.93	2.80 ± 0.96	0.687
apoA (g/L)	1.45 ± 0.30	1.41 ± 0.36	1.47 ± 0.24	0.517
apoB (g/L)	0.90 ± 0.30	0.89 ± 0.0.27	0.94 ± 0.28	0.647
FBG (mmol/L)	10.32 ± 4.31	10.87 ± 3.72	9.26 ± 3.31	0.068
HbA1c (%)	9.31 ± 2.36	9.86 ± 2.35	8.99 ± 2.47	0.130
WBC (∗10^9^/L)	6.72 ± 1.83	6.78 ± 1.77	6.66 ± 1.69	0.939
Neutrophil (∗10^9^/L)	4.49 ± 1.72	4.56 ± 1.64	4.64 ± 1.56	0.898
Lymphocyte (∗10^9^/L)	1.60 ± 0.64	1.67 ± 0.64	1.49 ± 0.53	0.272
NLR	3.46 ± 2.69	3.28 ± 2.27	3.72 ± 2.47	0.623
Hb (g/L)	137.23 ± 16.78	127.53 ± 18.48^∗∗^	122.35 ± 15.89^∗∗^	0.0001
TBIL (*μ*mol/L)	13.44 ± 5.68	12.13 ± 3.98	11.11 ± 3.84^∗^	0.037
DBIL (*μ*mol/L)	4.59 ± 2.07	4.45 ± 1.62	3.91 ± 1.65	0.124
IBIL (*μ*mol/L)	8.85 ± 4.00	7.69 ± 2.87	7.21 ± 2.90^∗^	0.037
GGT (U/L)	33.41 ± 27.91	29.75 ± 18.82	22.71 ± 12.98^∗^	0.038
Albumin (g/L)	42.53 ± 3.84	40.24 ± 4.81^∗∗^	40.93 ± 4.18	0.018
Creatinine (*μ*mol/L)	74.78 ± 21.13	79.85 ± 35.64	81.82 ± 43.10	0.622
eGFR (mL/min/1.73 m^2^)	90.56 ± 17.74	83.02 ± 25.71	77.92 ± 26.44^∗^	0.025
Urinary ACR (mg/g)	138.62 ± 52.50	367.52 ± 117.34^∗^	320.82 ± 97.40^∗^	0.019
UA (*μ*mol/L)	349.68 ± 100.07	348.31 ± 111.08	329.28 ± 103.72	0.527
Ca (mg/dL)^a^	9.04 ± 0.54	9.06 ± 0.47	9.05 ± 0.52	0.978
P (mmol/L)	1.15 ± 0.25	1.14 ± 0.26	1.16 ± 0.22	0.901
ALP (U/L)	75.61 ± 19.41	82.86 ± 31.36	89.11 ± 42.06	0.370
PTH (pg/mL)	44.77 ± 24.28	40.53 ± 19.60	50.00 ± 46.08	0.263
25(OH)D (ng/mL)	19.59 ± 8.93	20.17 ± 18.93	18.08 ± 11.50	0.720
TRACP-5b (mIU/mL)	1.50 ± 0.23	1.56 ± 0.19	1.75 ± 0.28	0.752
ABI	1.06 ± 0.12	1.05 ± 0.09	0.97 ± 0.21^∗^^,#^	0.002
VPT (V)	15.94 ± 8.41	16.71 ± 8.79	17.37 ± 8.81	0.699
Omentin-1 (ng/mL)	16.07 ± 2.06	17.97 ± 1.97	24.62 ± 2.68^∗^^,#^	0.024
OSTA	2.04 ± 0.38	−0.30 ± 0.31^∗∗^	−1.87 ± 0.38^∗∗^^,##^	0.0001
Bone metabolism				
LS BMD (g/cm^2^)	1.212 ± 0.133	1.032 ± 0.114^∗∗^	0.869 ± 0.121^∗∗^^,##^	0.0001
LS **T** score	0.73 ± 0.15	−0.94 ± 0.11^∗∗^	−2.34 ± 0.18^∗∗^^,##^	0.0001
FN BMD (g/cm^2^)	0.979 ± 0.101	0.787 ± 0.089^∗∗^	0.711 ± 0.097^∗∗^^,##^	0.0001
FN **T** score	−0.00 ± 0.10	−1.45 ± 0.09^∗∗^	−2.03 ± 0.15^∗∗^^,##^	0.0001
TH BMD (g/cm^2^)	1.048 ± 0.109	0.849 ± 0.095^∗∗^	0.750 ± 0.095^∗∗^^,##^	0.0001
TH **T** score	0.38 ± 0.11	−1.14 ± 0.09^∗∗^	−1.90 ± 0.14^∗∗^^,##^	0.0001
Prior fragility fractures	0 (0.00%)	0 (0.00%)	7 (12.96%)^∗∗^^,##^	0.0001
Macrovascular complications				
Hypertension (**n**, %)	33 (63.46%)	40 (60.61%)	39 (72.22%)	0.398
CHD (**n**, %)	2 (3.85%)	6 (9.09%)	6 (11.11%)	0.370
Stroke (**n**, %)	10 (19.23%)	21 (31.82%)	16 (29.63%)	0.284
PAD (**n**, %)	3 (5.77%)	2 (3.03%)	10 (18.52%) ^##^	0.008
Microvascular complications				
DN (**n**, %)	21 (40.38%)	32 (48.48%)	35 (64.81%)^∗^	0.037
DR (**n**, %)	3 (5.77%)	7 (10.61%)	6 (11.11%)	0.575
DPN (**n**, %)	16 (30.77%)	14 (21.21%)	14 (25.93%)	0.499
Hypoglycemic medication				
Metformin (**n**, %)	38 (73.08%)	46 (69.70%)	30 (55.56%)	0.124
Sulfonylurea (**n**, %)	29 (55.77%)	28 (42.42%)	18 (33.33%)	0.066
Alpha-glucosidase inhibitor (**n**, %)	9 (17.31%)	6 (9.09%)	10 (18.52%)	0.276
Insulin (**n**, %)	15 (28.85%)	26 (39.39%)	21 (38.89%)	0.434
Other (**n**, %)	3 (5.77%)	2 (3.03%)	4 (7.41%)	0.553

Data are mean ± SD. SD: standard deviation; BMI: body mass index; SBP: systolic blood pressure; DBP: diastolic blood pressure; TC: total cholesterol; TG: triglyceride; HDL-C: high-density lipoprotein cholesterol; LDL-C: low-density lipoprotein cholesterol; ApoA: apolipoprotein A; apoB: apolipoprotein B; FBG: fasting blood glucose; HbA1c: glycated hemoglobin A1c; WBC: white blood cell; NLR: neutrophil to lymphocyte ratio; Hb: hemoglobin; TBIL: total bilirubin; DBIL: direct bilirubin; IBIL: indirect bilirubin; GGT: gamma-glutamyltransferase; eGFR: estimated glomerular filtration rate; UA: uric acid; Ca: calcium ion; Ca (mg/dL)^a^ = serum calcium concentration (mg/dL) + 0.8 × [4.0 (g/dL) − serum albumin concentration (g/dL)]; P: phosphorus ion; ALP: alkaline phosphatase; PTH: parathyroid hormone; 25(OH)D: 25-hydroxyvitamin D; TRACP-5b: tartrate-resistant acid phosphatase 5b; ABI: ankle-brachial index; OSTA: osteoporosis self-assessment tool for Asians; LS: lumbar spine; FN: femoral neck; TP: total hip; BMD: bone mineral density; CHD: coronary heart disease; PAD: peripheral arterial disease; DN: diabetic nephropathy; DR: diabetic retinopathy; DPN: diabetic peripheral neuropathy; T2DM: type 2 diabetes mellitus. Versus the normal group: ^∗^**P** < 0.05; ^∗∗^**P** < 0.01. Versus the osteopenia group: ^#^**P** < 0.05; ^##^**P** < 0.01.

**Table 2:** Linear correlation analysis of variables associated with circulating omentin-1 in study subjects.

Variable	Simple			
	**r**	**P** value	Adjusted **r**	Adjusted **P** value
Age	-0.041	0.594	—	—
Sex	0.235	0.002	—	—
BMI	-0.131	0.087	—	—
Diabetic duration	0.027	0.727	0.039	0.505
SBP	-0.011	0.885	0.044	0.642
DBP	-0.042	0.582	-0.210	0.024
TC	0.092	0.229	-0.071	0.448
TG	-0.212	0.005	-0.216	0.020
HDL-C	0.368	0.0001	0.264	0.004
LDL-C	0.047	0.543	-0.095	0.309
apoA	0.283	0.0001	0.191	0.040
apoB	-0.022	0.772	-0.094	0.315
FBG	-0.142	0.064	-0.047	0.617
HbA1c	-0.041	0.590	-0.033	0.727
WBC count	-0.048	0.539	-0.096	0.256
Neutrophil count	0.018	0.819	-0.042	0.621
Lymphocyte count	-0.220	0.004	-0.205	0.014
NLR	0.202	0.009	0.084	0.318
Hb	-0.323	0.0001	-0.296	0.001
TBIL	-0.211	0.007	-0.147	0.115
DBIL	-0.283	0.0001	-0.133	0.225
IBIL	-0.151	0.056	-0.143	0.126
GGT	-0.264	0.001	-0.180	0.054
Serum albumin	-0.148	0.053	-0.148	0.112
Creatinine	-0.042	0.584	0.063	0.504
eGFR	-0.024	0.751	-0.044	0.636
Urinary ACR	0.257	0.001	0.119	0.155
UA	-0.190	0.012	-0.144	0.124
Ca	-0.007	0.930	-0.025	0.790
P	0.058	0.453	0.043	0.645
ALP	0.019	0.800	-0.074	0.427
PTH	0.109	0.156	-0.003	0.972
25(OH)D	-0.020	0.795	-0.113	0.226
TRACP-5b	-0.039	0.650	-0.102	0.274
ABI	0.001	0.987	0.067	0.429
VPT	0.002	0.979	-0.035	0.675
OSTA	-0.233	0.002	-0.291	0.0001
LS BMD	-0.185	0.015	-0.176	0.022
LS **T** score	-0.219	0.004	-0.181	0.018
FN BMD	-0.244	0.001	-0.215	0.005
FN **T** score	-0.214	0.005	-0.181	0.018
TH BMD	-0.243	0.001	-0.236	0.002
TH **T** score	-0.224	0.003	-0.210	0.006
Prior fragility fractures	0.202	0.008	0.227	0.003
Hypertension	-0.032	0.680	0.017	0.860
CHD	-0.037	0.628	-0.105	0.263
Stroke	-0.038	0.621	0.024	0.802
PAD	0.073	0.340	-0.031	0.714
DN	0.214	0.005	0.238	0.004
DR	0.159	0.037	0.224	0.016
DPN	0.012	0.872	-0.023	0.790

**Table 3:** Binary logistic regression analyses of variables contributing to osteoporosis in patients with T2DM.

Variables	Univariate analysis			Multivariate analysis		
	**B**	OR (95% CI)	**P**	**B**	OR (95% CI)	**P**
Omentin-1	0.031	1.031 (1.005-1.058)	0.020	0.034	1.035 (1.001-1.070)	0.046
Sex	2.365	10.649 (4.322-26.242)	0.0001	1.976	7.214 (2.387-21.804)	0.0001
Age	0.085	1.089 (1.038-1.142)	0.0001	0.077	1.080 (1.018-1.146)	0.010
Diabetic duration	0.009	1.009 (0.951-1.070)	0.766			
BMI	-0.107	0. 898 (0.803-1.005)	0.061			
TC	0.130	1.139 (0.848-1.529)	0.387			
TG	0.224	1.251 (0.955-1.640)	0.104			
HDL-C	-0.826	0.438 (0.142-1.355)	0.152			
LDL-C	0.156	1.169 (0.799-1.710)	0.421			
apoA	0.291	1.337 (0.316-5.650)	0.693			
apoB	0.401	1.494 (0.387-5.766)	0.561			
FBG	-0.075	0.928 (0.837-1.030)	0.159			
HbA1c	-0.056	0.946 (0.806-1.110)	0.493			
WBC	-0.019	0.981 (0.787-1.224)	0.866			
Neutrophil count	0.056	1.058 (0.834-1.342)	0.641			
Lymphocyte count	-0.332	0.717 (0.369-1.395)	0.327			
NLR	0.041	1.042 (0.895-1.214)	0.595			
Hb	-0.060	0.942 (0.914-0.971)	0.0001	-0.044	0.957 (0.922-0.993)	0.020
TBIL	-0.106	0.900 (0.821-0.985)	0.023			
GGT	-0.027	0.973 (0.950-0.997)	0.030			
Serum albumin	-0.100	0.905 (0.820-0.999)	0.047			
UA	-0.002	0.998 (0.994-1.002)	0.304			
Ca	0.029	1.030 (0.499-2.125)	0.937			
P	0.177	1.194 (0.232-6.147)	0.832			
ALP	0.015	1.015 (1.000-1.030)	0. 052			
PTH	0.004	1.004 (0.993-1.016)	0.477			
25(OH)D	-0.015	0.986 (0.948-1.024)	0.456			
TRACP-5b	0.095	1.100 (0.839-1.442)	0.489			
Hypertension	0.403	1.497 (0.659-3.400)	0.335			
CHD	1.139	3.125 (0.601-16.249)	0.176			
Stroke	0.570	1.768 (0.716-4.366)	0.216			
PAD	1.312	3.712 (0.960-14.361)	0.057			
DN	1.000	2.719 (1.238-5.972)	0.013			
DR	0.714	2.042 (0.483-8.634)	0.332			
DPN	-0.239	0.788 (0.338-1.837)	0.580			

Beta is the standardized coefficient and measures the influence of each variable on osteoporosis; OR is the odds ratio and refers to the risk of osteoporosis.

**Table 4:** Circulating omentin-1 level and its association with the increased presence of osteoporosis in men and women, respectively.

Variables	Men (**n** = 89)	Women (**n** = 83)
Omentin-1 (ng/mL)	15.52 ± 1.39	23.74 ± 2.18^∗^
Unadjusted OR	1.016	1.102
95% CI	0.978-1.056	1.012-1.201
**P** value	0.419	0.026
Multivariate OR	1.042	1.069
(95% CI)	0.932-1.165	1.003-1.139
**P** value	0.469	0.041

OR is the odds ratio and refers to the risk of osteoporosis. The multivariate model is adjusted for age, diabetic duration, BMI, TC, TG, HDL-C, LDL-C, apoA, apoB, FBG, HbA1c, WBC, NLR, neutrophil count, lymphocyte count, Hb, TBIL, GGT, serum albumin, UA, Ca, P, ALP, PTH, 25(OH)D, TRACP-5b, prevalence of hypertension, CHD, stroke, PAD, DN, DR, and DPN vs. men; ^∗^*P* < 0.01.

## Data Availability

The data used to support the findings of this study are available from the corresponding author upon request.
